# COVID-19 vaccination hesitance and adverse effects among US adults: a longitudinal cohort study

**DOI:** 10.3389/fepid.2024.1365090

**Published:** 2024-07-16

**Authors:** M. Abdelmasseh, A. Cuaranta, A. Iqbal, V. Kadiyala, J. Willis, A. Gorka, E. Thompson, R. Finley, B. Payne, J. Sanabria

**Affiliations:** ^1^ Department of Surgery, Marshall University School of Medicine, Huntington, WV, United States; ^2^ Marshall Institute for Interdisciplinary Research (MIIR), Marshall University, Huntington, WV, United States; ^3^ Department of Informatics and Biostatistics, Marshall University School of Medicine, Huntington, WV, United States

**Keywords:** COVID-19, SARS-COV2, vaccine, survey, hesitancy, pandemic, adverse events

## Abstract

**Introduction:**

Although Coronavirus disease 2019 (COVID-19) vaccination is critical to control its spread, vaccine hesitancy varies significantly among the United States population; moreover, some vaccine recipients experienced various adverse effects. We aim to assess the impact of COVID-19 vaccine hesitancy in a university-affiliated community, the factors affecting participants’ decisions, and their adverse effects.

**Methods:**

A pre-vaccination online Institutional Review Board IRB-approved survey was emailed in Nov/Dec 2020, 2 months before the implementation of state-policy protocols for COVID-19 vaccination. A post-vaccination survey was emailed in May/June 2021, two months after protocol execution. A third follow-up survey was sent in Nov/Dec 2021, and a fourth was sent in June/July 2022. The study population included three groups of adult participants: university students, faculty, and staff-(MS), university health system patients-(MP), and Cancer Center patients-(MCP). The study was designed as a longitudinal cohort study. Statistical analyses were performed using SPSS.

**Results:**

With a combined response rate of 26% (40,578/157,292) among the four surveys, 15,361 participants completed the first survey (MS = 4,983, MP = 9,551, and MCP = 827). 2/3 of participants (63.5%) were willing to get vaccinated, with a significant difference in acceptance among groups, MS:56.6%, MP:66.2%, and MCP:71.6% (*p *< 0.05). Vaccine acceptance rates reached 89% in the second survey after the vaccine's approval, with a lower acceptance rate of MS:84.6% than with MP:90.74% and MCP:92.47% participants (*p *< 0.05). Safety and effectiveness concerns were the main factors affecting participants’ decisions in all the first three surveys; however, participants reported these concerns decreased between pre-vaccination, post-vaccination, and follow-up surveys with 87%, 56%, and 46%, respectively(*p *< 0.05). More than two-thirds of the participants (70%) reported having either minor/moderate symptoms (61.6%) or major symptoms (8.6%) after getting some of the vaccine doses (*p* < 0.05).

**Conclusion:**

The hesitance of COVID-19 vaccination was associated with concerns regarding its safety and efficacy. Vaccine acceptance rose higher than expected after protocol execution, likely due to continuous education, whereas safety and efficacy remain factors hindering vaccine acceptance. Continuous education focusing on safety and efficacy of the vaccine can reduce vaccine hesitancy and raise the rates of vaccination.

## Introduction

The COVID-19 (Coronavirus disease 2019) pandemic has impacted nearly every country, infected around 775 million people, and has surpassed 7 million deaths worldwide (1). The Global and United States (US) economies have been heavily affected by a viral disease caused by the severe acute respiratory syndrome coronavirus 2 (SARS-COV2), overwhelming healthcare facilities (2, 3). Despite the Centers for Disease Control and Prevention (CDC) guidelines from medical experts and healthcare advisors, the preventive measures of wearing face masks, washing hands frequently, and keeping social distancing (4) have not rescinded the spread of SARS-COV2 throughout the world. With the emergence of new and more contagious variants such as Delta and Omicron, the spread of infection was reaching unprecedented rates worldwide (5–9). The need to raise the vaccination acceptance rates is always important to control infections and relieve the pandemic's burden.

From the initial reported infection of COVID-19 in early December 2019, it took a full year for the world to develop the first vaccine, which was achieved in December 2020 (10). The United Kingdom was the first country to grant emergency authorization for the public administration of the mRNA-based vaccine, followed by the United States (11, 12). The United States Food and Drug Administration (US FDA) authorized the Pfizer-BioNTech COVID-19 vaccine for children aged 12–15 by May 2021. In addition, the US FDA gave the vaccine full authorization in August 2021 (13). Moreover, in October 2021, the US FDA approved vaccinations for children five years or older (14). More than 14 billion doses of the vaccine were administered worldwide; however, only 70.6% of the world population has received at least one dose of a COVID-19 vaccine. In the US, more than 712 million doses were administered, and nearly 70% of the population were vaccinated with a complete primary series of COVID-19 vaccination (10, 15). Governments and industries have come together to overcome the unprecedented challenges of massive distribution logistics. Nevertheless, health policymakers, scientists, and providers are still facing an uphill battle with strategies to increase acceptance of the COVID-19 vaccine, mainly when some vaccine recipients reported experiencing adverse effects post-vaccination. However, adverse event rates were reported more in their phase 3 trials (16), increasing the challenges that improving vaccination rates may face.

Since the conception of the world's first vaccine for smallpox in 1798 (17, 18), an evolution in the movement of acceptance *vs.* hesitancy has grown based on vaccine safety and efficacy and its inherent adverse effects (19, 20). In 2015, a group of experts from the World Health Organization (WHO) defined “vaccine hesitancy” as the delay in acceptance or refusal of vaccination despite vaccination services’ availability (21). In 2019, vaccine hesitancy was ranked as one of the top ten threats to global health (22). Vaccine hesitancy and uncertainty have become significant hurdles for reaching desirable societal immunity against targeted diseases in many countries (23, 24). In cooperation with policymakers and other stakeholders, governments and health societies are required to improve the process of providing the necessary information about the vaccine and enhance the understanding and importance of immunization to reach the immunity of the community. The present study aimed to assess the COVID-19 vaccination acceptance and hesitance rates and the factors impacting people's decisions regarding vaccination, besides evaluating the adverse effects participants might have experienced post-vaccination.

## Materials and methods

### Study design

The study was designed as a longitudinal cohort study over two years. A pre-vaccination online survey was developed by (Qualtrics® Services), linked to the participants’ emails, and sent upon Institutional Review Board (IRB) approval. The survey was emailed (Supplementary Appendix 1) in Nov/Dec 2020, 2-months before state-policy protocols implementation for COVID-19 vaccine administration. It intended to determine the participants’ acceptance of vaccination. A second post-vaccination survey was emailed (Supplementary Appendix 2) in May/June 2021, 2-months after protocols were executed. The post-vaccination survey was sent weekly with a one-month enrollment period to determine the actual vaccination rates. A third follow-up survey was emailed (Supplementary Appendix 3) in Nov/Dec 2021, 5-months after the second survey. It aimed to determine the reasons affecting participants’ decisions on whether they changed their minds before and after vaccine approval. A fourth and final survey (Supplementary Appendix 4) was sent in June/July 2022 to define adverse events participants may have experienced.

### Study population

Individual emails were gathered from a central University and Health System warehouse and divided into three groups: (i) University faculty, students, and staff (University Students and Staff/MS); (ii) University School of Medicine affiliated Health System registered patients (General Patients/MP), and (iii) University School of Medicine affiliated Comprehensive Cancer Center registered cancer patients (Cancer Patients/MCP). An individual survey was sent weekly for three weeks with a one-month enrollment period. The software identified duplicates in each survey, which were deleted so that one individual had only one response to each of the four surveys.

The surveys were anonymous to the study team but had a unique IP address. The number of questions ranged from six questions in the first survey, seven to nine questions in the second, eight to ten in the third survey, and fourteen to twenty-three in the fourth (see Supplementary Appendix 1–S4). Each survey contained demographic questions about gender/sex, ethnicity/race, education level, and age category. Tacit consent was given by moving forward from the introductory survey page.

### Statistical analysis

All variables were categorical and described as frequencies and percentages. For predictor variables, we generally used gender, age category, ethnicity, and education and occasionally included trusted sources of information or vaccine type. Outcome variables varied for each survey (i.e., Survey 1—the outcome is whether the respondent will get vaccinated, Survey 2—the outcome is whether the respondent received at least one dose of a vaccine, Survey 3—the outcome is whether the respondent changed their mind about getting vaccinated, Survey 4—the outcome is whether the respondent experienced side effects/symptoms following any of the vaccine doses they received).

Univariate and Multivariate analyses were performed using ordinal and binomial logistic regressions. Area under the curve (AUC) analyses of receiver operating characteristic (ROC) curves were examined for the logistic regression models to determine prediction accuracy. Results were expressed in terms of probability (*p *< 0.05) and odds ratio estimates with 95% confidence intervals. For logistic regressions, the *p*-value measures the statistical significance of the estimated coefficients for the predictor variables and tests the null hypothesis that there is no relationship between that predictor and the outcome variables (i.e., the parameter coefficient is equal to 0). Statistical analyses were performed using SPSS version 28 (SPSS-IBM Inc., Chicago, IL, USA).

## Results

Out of the 157,292 surveys distributed to all participants, the response rate exhibited considerable variations across the four surveys. The first survey achieved a response rate of 10% (*n* = 15,361), the second survey 7.3% (*n* = 11,539), and both the third and fourth surveys 4.3% (*n* = 6,902 and *n* = 6,776, respectively). Notably, significant differences in response rates among participant groups were observed for each survey (Supplementary Table S1). While the response rate was higher in the MS group (27.4% and 10.5% for the first and fourth surveys, respectively), the MP group showed lower response rates (7.5% and 2.7%). As expected, participants’ demographics differed among groups and by survey (Table 1). The MS group was younger when compared to the MP and the MCP groups (*p *< 0.05). The education degree was lower in the MS group compared to the MP and MCP groups (*p *< 0.05). In contrast, the sex distribution at a ratio of 3♀:2♂ among groups was similar for the four surveys. Most of the responders were white, with some diversity in the MS group (*p *< 0.05).

**Table 1:** Participants’ demographic distribution from survey 1(Pre-vaccination), Survey 2 (post-vaccination), Survey 3 (follow-up), and Survey 4 (Side effects).

Survey 1								
	MS		MP		MCP		Total	
	Total respondents *n*	Willing to get vaccinated *n* (%)	Total respondents *n*	Willing to get vaccinated *n* (%)	Total respondents *n*	Willing to get vaccinated *n* (%)	Total respondents *n*	Willing to get vaccinated *n* (%)
	(*N* = 4,983)	2,807 (56.6)	(*N* = 9,551)	6,432 (66.2)	(*N* = 827)	598 (71.6)	(*N* = 15,361)	9,836 (63.5)
Age								
18–24	2,524	1,507 (59.7)	408	252 (61.7)	8	5 (62.5)	2,933	1,766 (60.2)
25–34	733	405 (55.2)	1,036	551 (53.2)	22	10 (45.4)	1,791	965 (53.9)
35–44	440	253 (57.5)	1,517	845 (55.7)	67	34 (50.7)	2,024	1,131 (55.9)
45–54	450	233 (51.7)	1,555	913 (58.7)	96	49 (51)	2,101	1,195 (56.9)
55–64	358	221 (61.7)	2,003	1,418 (70.8)	189	144 (76.1)	2,550	1,785 (70)
65 or older	243	197 (81)	2,907	2,465 (84.8)	426	362 (84.9)	3,576	3,022 (84.5)
Sex								
Male	1,603	1,100 (68.6)	3,337	2,613 (78.3)	278	240 (86.3)	5,218	3,950 (75.7)
Female	3,133	1,707 (54.5)	6,071	3,819 (62.9)	525	359 (68.3)	9,729	5,886 (60.5)
Race								
White	4,197	2,497 (59.5)	8,996	6,189 (68.8)	783	590 (75.4)	13,776	9,271 (67.3)
AA^a^	180	78 (43.3)	154	84 (54.5)	5	4 (80)	339	166 (48.9)
Asian	164	120 (73.1)	76	61 (80.2)	4	1 (25)	244	182 (74.5)
NA^b^	15	7 (46.6)	26	13 (50)	4	4 (100)	45	24 (53.3)
Others	177	108 (61)	134	80 (59.7)	8	1 (12.5)	319	189 (59.2)
Education								
HS^c^ diploma	545	312 (57.2)	920	618 (67.2)	109	74 (67.8)	1,574	1,004 (63.8)
In college	1,724	1,014 (58.8)	1,795	1,221 (68)	166	132 (79.5)	3,685	2,366 (64.2)
Associate degree	260	106 (40.7)	1,166	667 (57.2)	100	71 (71)	1,526	844 (55.3)
Bachelor's degree	947	545 (57.6)	2,359	1,637 (69.4)	154	114 (74)	3,460	2,297 (66.4)
Master's degree	654	407 (62.3)	1,837	1,350 (73.5)	138	110 (79.7)	2,629	1,869 (71.1)
Doctorate degree	525	390 (74.2)	653	539 (82.6)	46	36 (78.2)	1,224	966 (78.9)

^a^ AA, African American.

^b^ NA, Native American.

^c^ HS, High School. The groups of participants are University faculty, students, and employees (MS), University-affiliated Health System registered patients (MP), and University-affiliated Comprehensive Cancer Center registered cancer patients (MCP).

**Table:** 

Survey 2								
	MS		MP		MCP		Total	
	Total respondents *n*	Vaccinated respondents *n* (%)	Total respondents *n*	Vaccinated respondents *n* (%)	Total respondents *n*	Vaccinated respondents *n* (%)	Total respondents *n*	Vaccinated respondents *n* (%)
	(*N* = 3,587)	2,891 (85)	(*N* = 7,189)	6,294 (90.3)	(*N* = 763)	677 (92.5)	(*N* = 11,539)	11,089 (88.6)
Age								
18–24	1,851	1,455 (78.6)	250	221 (88.4)	3	3 (100)	2,104	1,679 (79.8)
25–34	423	368 (86.9	700	593 (84.7)	19	15 (78.9)	1,142	976 (85.4)
35–44	335	314 (93.7)	992	83 (84.4)	53	43 (81.1)	1,380	1,195 (86.5)
45–54	343	319 (93)	1,207	1,047 (86.7)	90	79 (87.7)	1,640	1,445 (88.1)
55–64	299	285 (95.3)	1,542	1,429 (92.6)	152	147 (96.7)	1,993	1,861 (93.3)
65 or older	158	152 (96.2)	2,559	2,194 (85.7)	413	395 (95.6)	2,830	2,741 (96.8)
Sex								
Male	1,143	985 (86.1)	2,553	2,341 (91.6)	268	253 (94.4)	3,964	3,579 (90.2)
Female	2,266	1,906 (84.1)	4,397	3,953 (89.9)	462	424 (91.4)	7,125	6,283 (88.1)
Race								
White	3,054	2,584 (84.6)	6,582	5,993 (91)	700	654 (93.4)	10,336	9,231 (89.3)
AA^a^	122	104 (85.2)	140	133 (95)	12	11 (91.6)	274	248 (90.5)
Asian	108	105 (97.2)	65	65 (100)	6	6 (100)	179	176 (98.3)
NA^b^	5	3 (60)	30	25 (83.3)	0	0	35	28 (80)
Others	95	81 (85.2)	96	76 (79.1)	9	6 (66.6)	200	163 (81.5)
Education								
HS^c^ diploma	602	460 (76.4)	739	673 (91)	113	106 (93.8)	1,454	1,239 (85.2)
In college	1,152	931 (80.8)	1,230	1,094 (88.9)	120	111 (92.5)	2,502	2,136 (85.3)
Associate degree	183	141 (77)	782	680 (86.9)	77	68 (88.3)	1,042	889 (85.3)
Bachelor's degree	562	497 (88.4)	1,706	1,565 (91.7)	147	136 (92.5)	2,415	2,198 (91)
Master's degree	470	447 (95.1)	1,480	1,386 (93.6)	151	142 (94)	2,101	1,975 (94)
Doctorate degree	378	369 (97.6)	515	492 (95.5)	55	54 (98.1)	948	915 (96.5)

^a^ AA, African American.

^b^ NA, Native American.

^c^ HS, High School. The groups of participants are University faculty, students, and employees (MS), University-affiliated Health System registered patients (MP), and University-affiliated Comprehensive Cancer Center registered cancer patients (MCP).

**Table:** 

Survey 3								
	MS		MP		MCP		Total	
	Total respondents *n*	Vaccinated respondents *n* (%)	Total respondents *n*	Vaccinated respondents *n* (%)	Total respondents *n*	Vaccinated Respondents *n* (%)	Total respondents *n*	Vaccinated respondents *n* (%)
	(*N* = 2,995)	2,507 (83.7)	(*N* = 3,485)	2,953 (84.7)	(*N* = 422)	386 (91.4)	(*N* = 6,902)	5,843 (84.7)
Age								
18–24	1,522	1,212 (79.6)	171	143 (83.6)	4	3 (75)	1,967	1,357 (69)
25–34	426	371 (87)	359	271 (75.5)	5	3 (60)	790	645 (81.6)
35–44	349	310 (88.8)	522	395 (75.6)	31	24 (77.4)	902	729 (80.8)
45–54	306	268 (87.5)	567	463 (81.6)	45	39 (86.6)	918	769 (83.8)
55–64	242	212 (87.6)	754	657 (87.2)	100	93 (93)	1,096	962 (87.8)
65 or older	150	144 (96)	1,112	1,033 (92.9)	237	226 (95.3)	1,499	1,403 (93.6)
Sex								
Male	1,004	832 (82.8)	1,298	1,120 (86.3)	140	131 (93.5)	2,442	2,083 (85.3)
Female	1,991	1,675 (84.1)	2,187	1,833 (83.8)	282	255 (90.4)	4,460	3,760 (84.3)
Race								
White	2,719	2,269 (83.4)	3,316	2,819 (85)	406	371 (91.3)	6,437	5,459 (84.8)
AA^a^	99	83 (83.8)	54	49 (90.7)	7	7 (100)	160	139 (86.8)
Asian	79	74 (93.6)	29	28 (96.5)	3	3 (100)	111	105 (94.5)
NA^b^	7	6 (85.7)	12	10 (83.3)	1	1 (100)	20	17 (85)
Others	79	68 (86)	53	37 (69.8)	4	4 (100)	136	109 (80.1)
Education								
HS^c^ diploma	251	196 (78)	302	256 (84.8)	55	49 (89)	608	501 (82.4)
In college	1,154	920 (79.7)	577	481 (83.3)	60	52 (86.6)	1,791	1,453 (81.1)
Associate degree	173	139 (80.3)	355	285 (80.2)	38	31 (81.5)	566	454 (80.3)
Bachelor's degree	621	536 (86.3)	917	775 (84.5)	94	91 (96.8)	1,632	1,402 (85.9)
Master's degree	439	400 (91.1)	800	693 (86.6)	101	99 (98)	1,340	1,191 (88.9)
Doctorate degree	305	288 (94.4)	321	297 (92.5)	45	43 (95.5)	671	627 (93.5)

^a^ AA, African American.

^b^ NA, Native American.

^c^ HS, High School. The groups of participants are University faculty, students, and employees (MS), University-affiliated Health System registered patients (MP), and University-affiliated Comprehensive Cancer Center registered cancer patients (MCP).

**Table:** 

Survey 4								
	MS		MP		MCP		Total	
	Total respondents *n*	Vaccinated respondents *n* (%)	Total respondents *n*	Vaccinated respondents *n* (%)	Total respondents *n*	Vaccinated respondents *n* (%)	Total respondents *n*	Vaccinated respondents *n* (%)
	(*N* = 1,917)	1,705 (93.6)	(*N* = 4,400)	3,982 (93.4)	(*N* = 459)	411 (94.1)	(*N* = 6,776)	6,098 (93.3)
Age								
18–24	743	667 (89.7)	114	113 (99.1)	4	4 (100)	861	784 (91)
25–34	265	246 (92.8)	440	403 (91.6)	7	6 (85.7)	712	655 (91.9)
35–44	236	227 (96.1)	667	604 (90.5)	35	26 (74.3)	938	857 (91.3)
45–54	231	224 (96.9)	719	659 (91.6)	55	52 (94.5)	1,005	935 (93)
55–64	215	206 (95.8)	887	816 (91.9)	91	85 (93.4)	1,193	1,107 (92.7)
65 or older	141	140 (99.2)	1,455	1,395 (95.8)	251	241 (96)	1,847	1,776 (96.1)
Sex								
Male	552	514 (93.1)	1,430	1,346 (94.1)	151	147 (97.3)	2,133	2,007 (94)
Female	1,274	1,191 (93.4)	2,842	2,636 (92.7)	289	264 (91.3)	4,405	4,091 (92.8)
Race								
White	1,675	1,568 (93.6)	4,107	3,828 (93.2)	427	400 (93.6)	6,209	5,796 (93.3)
AA^a^	61	53 (86.8)	66	64 (96.9)	7	7 (100)	134	124 (92.5)
Asian	45	45 (100)	27	27 (100)	3	3 (100)	75	75 (100)
NA^b^	4	4 (100)	6	6 (100)	2	2 (100)	12	12 (100)
Others	38	35 (92.1)	66	57 (86.3)	3	1 (33.3)	107	93 (86.9)
Education								
HS^c^ diploma	220	191 (86.8)	418	383 (91.6)	54	50 (92.5)	692	624 (90)
In college	487	438 (89.9)	686	638 (93)	81	77 (95)	1,254	1,153 (91.9)
Associate degree	108	101 (93.5)	479	440 (91.8)	60	48 (80)	647	589 (91)
Bachelor's degree	377	353 (93.6)	1,111	1,035 (93.1)	76	70 (92.1)	1,564	1,458 (93.2)
Master's degree	317	308 (97.1)	972	919 (94.5)	99	94 (95)	1,388	1,321 (95.1)
Doctorate degree	277	276 (99.6)	332	319 (96)	35	34 (97.1)	644	629 (97.6)

^a^ AA, African American.

^b^ NA, Native American.

^c^ HS, High School. The groups of participants are University faculty, students, and employees (MS), University-affiliated Health System registered patients (MP), and University-affiliated Comprehensive Cancer Center registered cancer patients (MCP).

**Figure 1:**
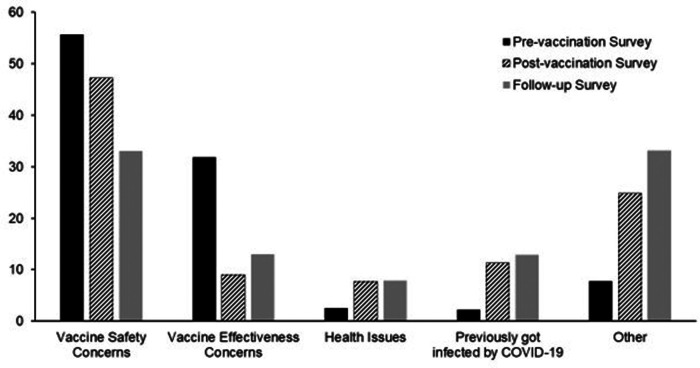
Factors affecting COVID-19 vaccination hesitance in survey 1 (Pre-vaccination), survey 2 (post-vaccination), and survey 3 (follow-up). Safety and efficacy concerns were the main factors affecting participants’ decisions in all three surveys: pre-vaccination (87%), post-vaccination (56%), and follow-up surveys (46%, *p* < 0.05).

The participant proportion willing to get vaccinated upon the vaccine's approval was 63.5%, ranging from 56.6% in the MS group to 66.2% and 71.6% in the MP and MCP groups, respectively (MS vs. MP and MCP, *p *< 0.05). Vaccine acceptance rates for each group were higher, reaching 88.6% (85%, 90.3%%, and 92.5% for MS, MP, and MCP groups, respectively) after the vaccine's approval by the FDA. The follow-up survey (survey 3) indicated that 17.2% of the participants accepted vaccination after initially refusing it. Participants reported health workers, family, and friends as the main trusted sources influencing their decisions (Supplementary Figure S1). Safety and efficacy concerns were the main factors affecting participants’ decisions in all three surveys: pre-vaccination (survey 1, 87%), post-vaccination (survey 2, 56%), and follow-up (survey 3, 46%, Figure 1, *p *< 0.05).

Most participants (70%) reported an adverse event from vaccination, and one out of 10 sought medical evaluation. Minor/moderate symptoms (61.6%) consisted of tiredness/fatigue and pain at the site of injection, observed more frequently after the second dose (36.1% and 28.8% for the tiredness/fatigue and pain at the site of injection, respectively). Major symptoms (8.6%) included anxiety and high-grade fever, which were the most reported after vaccination, mainly also after the second dose (3.9% and 3.7% for anxiety and high-grade fever, respectively, Table 2).

**Table 2:** Participants’ adverse events after any dose of an approved COVID-19 vaccine.

	First dose		Second dose		Third dose		Fourth dose	
	(*N* = 2,443)		(*N* = 3,011)		(*N* = 1,740)		(*N* = 472)	
	*n*	%	*n*	%	*n*	%	*n*	%
A) Minor/moderate symptoms								
Low-grade fever of 99.5°F–100.3°F (37.5°C–38.3°C)	874	13.8	1,155	18.2	618	9.7	119	1.88
Tiredness/fatigue	1,814	28.6	2,289	36.1	1,260	19.9	291	4.59
Pain at the injection site	1,638	25.8	1,827	28.8	1,070	16.9	303	4.78
Inflammation or swelling at the injection site	655	10.3	746	11.8	417	6.6	101	1.59
Headache	1,017	16.0	1,343	21.2	706	11.1	137	2.16
Joints pain	727	11.5	1,015	16.0	556	8.8	102	1.61
Muscular pain	837	13.2	1,105	17.4	641	10.1	113	1.78
Skin rash	109	1.7	111	1.8	68	1.1	10	0.16
Irritation or itchy skin	128	2.0	143	2.3	77	1.2	21	0.33
Diarrhea	107	1.7	275	4.3	110	1.7	31	0.49
Nausea/vomiting	235	3.7	296	4.7	142	2.2	23	0.36
Other	307	4.8	369	5.8	186	2.9	47	0.74
B) Major symptoms								
Anaphylaxis	7	0.1	3	0.0	0	0.0	0	0.0
Anxiety	181	2.9	248	3.9	103	1.6	18	0.3
High-grade fever >100.3°F (>38.3°C)	167	2.6	234	3.7	106	1.7	21	0.3
Dyspnea	117	1.8	146	2.3	64	1.0	10	0.2
Loss of consciousness	12	0.2	14	0.2	5	0.1	2	0.0
Blood clots	13	0.2	15	0.2	4	0.1	2	0.0
Seizure	6	0.1	5	0.1	2	0.0	2	0.0
Myocarditis	22	0.3	22	0.3	15	0.2	1	0.0
Hospitalization	13	0.2	25	0.4	15	0.2	1	0.0
Other	131	2.1	161	2.5	71	1.1	25	0.4

**Figure 2:**
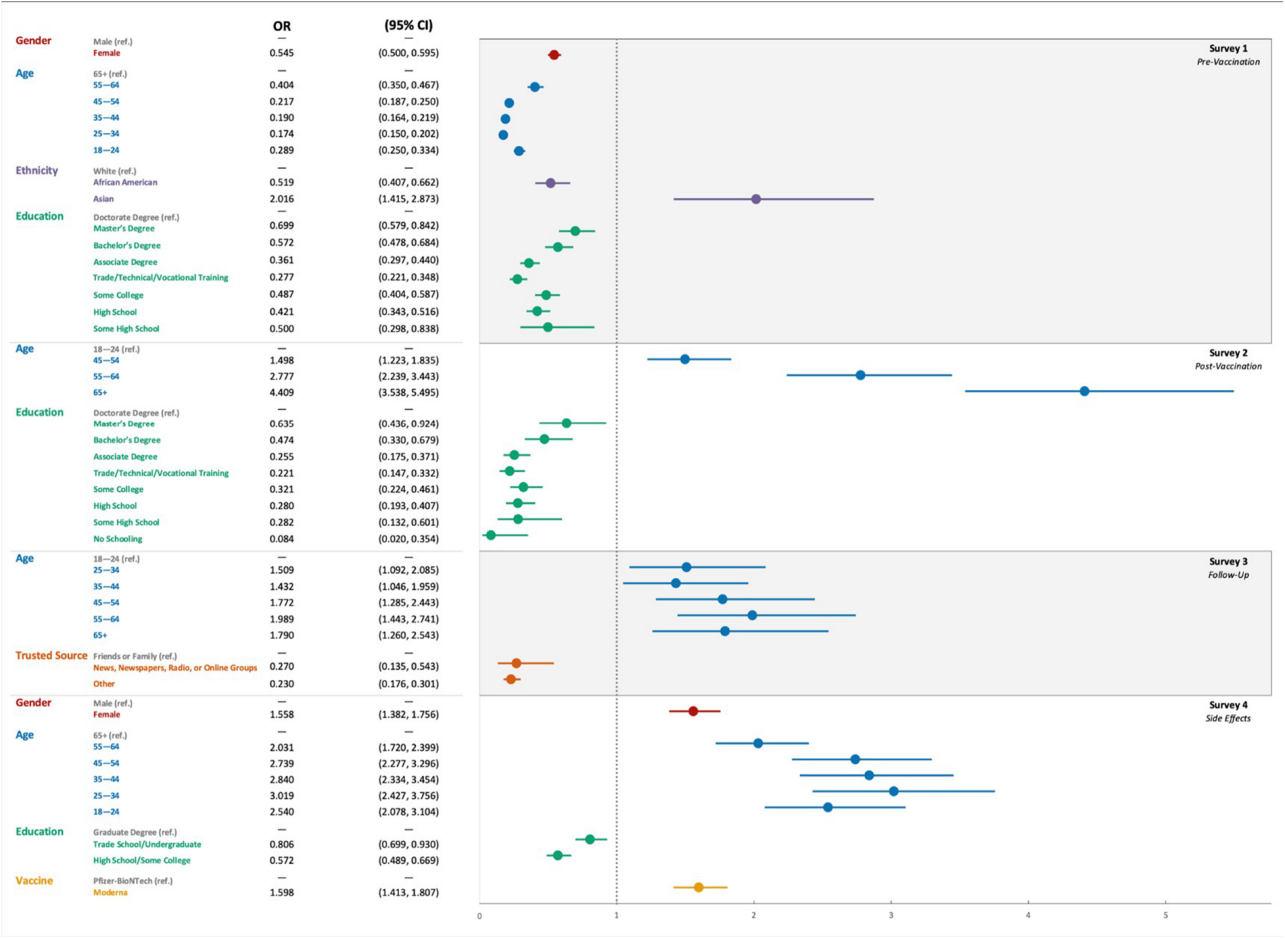
The odds ratio using multivariate analyses for the variables affecting the COVID-19 vaccination hesitance and side effects in the four consecutive surveys: survey 1 (Pre-vaccination), Survey 2 (post-vaccination), Survey 3 (follow-up), and Survey 4 (adverse events).

Univariate and multivariate analyses were performed to determine the factors associated with vaccine hesitance before and after vaccination (Figure 2). On multivariate analyses, age (>44 years old), race (white), gender, and education (bachelor's degree or higher) were factors that statistically held a significant association with participants’ vaccine acceptance prior to vaccination (*p *< 0.05). After vaccination, age (> 34 years old) and level of education (bachelor's degree or higher) remained significant (*p *< 0.01). In contrast, age and trusted source were the only factors significantly associated with the change of mind for vaccine hesitance (*p *< 0.05). Both univariate and multivariate analyses showed that age, gender, level of education, and the type of the vaccine were statistically significant in predicting the adverse events that the participants experienced after any dose (Supplementary Table S2, *p *< 0.01).

## Discussion

The need for an effective and safe vaccine to relieve the pandemic's burden of COVID-19 infection has proven to be persistent, especially with frequent virus mutations. The present study focuses on the status of vaccine acceptance among US adults, factors associated with hesitancy and their decisions, and vaccine adverse events. To the best of our knowledge, this study represents one of the largest of its kind to assess COVID-19 vaccination hesitancy. Overall, two-thirds of the study population were willing to get vaccinated, and their main concerns were the safety and efficacy of the vaccine; the percentage increased from 64% to 89% after the vaccine's approval. Moreover, around 17% of the participants eventually changed their minds and accepted the vaccines. Nevertheless, 70% of the participants reported, mainly after the second dose, either minor to moderate symptoms (61.6%) or major symptoms (8.6%) after getting vaccinated.

Prior studies showed the COVID-19 vaccine hesitance as a real impediment to achieving the immunity required to reach herd immunity (25–27). A global survey from 19 countries (*n* = 13,426) showed a pre-vaccination acceptance rate of 71.5% (28). Nonetheless, the study was skewed from outlier higher rates from two Asian countries (in the range of 85%), which also recorded very high trust in government health recommendations. A study of American US adults (*n* = 1,056) showed a lower acceptance rate (49%) (29). The hesitance to vaccination varied significantly depending on the other populations surveyed (50%–73%) (30–32).

Concerns with the vaccines pre- and post-approval were similar: safety and efficacy. Although the safety and efficacy profiles were proved to be favorable for most of the vaccines (33–35) and against recent variants (36, 37), adverse events varied in rate and severity among different populations (38–40). Most of the adverse events reported were mild to moderate (the most common were pain at the injection site, fatigue, and headache) (41, 42). Our study concurred with the published literature. Previous studies also showed that healthcare workers were the most trusted source of information regarding COVID-19 vaccines. People tend to trust their healthcare providers rather than the media. Our study proved similar results where nearly half of the participants reported trusting the health workers as their primary source of information.

Although studies have shown a solid scientific base for the needed increase in vaccination rate, substantial efforts are essential by governments and public health officials to enhance vaccine acceptance. Approaching some of the factors that play into the individual decision may pave the way for a paucity of vaccine hesitance. The present study found a significant association between COVID-19 vaccine acceptance and age, gender, race, and education level. While age, gender, and race were variables with no room for modification, general education on public media may dissipate community concerns about the safety and efficacy of the COVID-19 vaccine. Processes and results shown in an easily interpretable and transparent manner may go a long way in trust. A clear and logical approach to actual, theoretical, and fictional implications of an RNA-based vaccine will mitigate differences between those who accept and those who refuse vaccination, avoiding confusion from contradictory facts, especially in the presence of an intense media-originated by anti-vaccination activists (43).

The present study represents the largest in the US to assess COVID-19 vaccination acceptance, with a combined 40,578 responses. Even though there was a low response rate, the study had an adequate sample size; however, race predominance is a limitation to the interpretation and extrapolation of the findings. Moreover, our surveys were sent electronically through email to the participants, which excluded those who didn't have access to emails. In addition, self-reported data are subject to a potential lack of validity and reliability as they may be affected by different biases.

## Conclusion

The present study is intended to provide a comprehensive image of the COVID-19 vaccines, assess their acceptance, identify factors that are associated with the individual decisions in the US, and assess their safety outcomes. A low vaccine acceptance rate was associated with a high degree of concern (89%) regarding its efficacy and safety before the vaccines’ approval. Nevertheless, vaccine acceptance rose higher than expected after protocol execution, likely due to continuous education. Safety and efficacy remain factors hindering vaccine acceptance. Most of the reported vaccine adverse events were mild to moderate, with minimal need for medical consulting. Continuous education concerning the importance of vaccination, along with discussing and proving the vaccine's safety and efficacy, can be the main tools to decrease the rates of vaccine hesitancy.

## Data Availability

The raw data supporting the conclusions of this article will be made available by the authors, without undue reservation.
