# Supplementary material for: COVID-19 vaccination hesitance and adverse effects among US adults: a longitudinal cohort study
Source: Front Epidemiol. 2024 Jul 16;4:1365090. doi: 10.3389/fepid.2024.1365090 (PMC11286423; doi:10.3389/fepid.2024.1365090)
Supplement: Supplementary file 1 [file Datasheet1.docx]

**Appendix 1:** Pre-vaccination survey questions (Survey 1)

Q1 When a vaccine against COVID-19 becomes approved and available, how likely would you be to get vaccinated?

- Extremely likely
- Somewhat likely
- Neither likely nor unlikely
- Somewhat unlikely
- Extremely unlikely

Q2 What are the factors that may affect your decision of getting vaccinated? (Please choose all that apply)

- Vaccine safety concerns
- Vaccine effectiveness concerns
- Previously got infected by COVID-19
- Prefer to get infected (it is safer than getting vaccinated)
- Other, please specify below _______________________________
- None of the above

Q3 Gender

- Male
- Female

Q4 Age Category

- 18 - 24
- 25 - 34
- 35 - 44
- 45 - 54
- 55 - 64
- 65 or older

Q5 Ethnicity

- White
- African American
- Asian
- Native American
- Other

Q6 Education: What is the highest degree or level of education completed?

- No schooling completed
- Some high school, no diploma
- High school graduate, diploma or equivalent (ex: GED)
- Some college credit, no degree
- Trade/technical/vocational training
- Associate degree
- Bachelor's degree
- Master's degree
- Doctorate degree.

**Appendix 2:** Post-vaccination survey questions (Survey 2)

Q1 Have you received the COVID-19 vaccine, either one dose or 2 doses?!

- Yes
- No

Q2 If yes, Which vaccine did you get?

- Pfizer-BioNTech
- Moderna
- Johnson & Johnson / Janssen
- One dose from a company and the second from another
- Other, please specify below _____________________________________

Q3 How many doses did you get so far?

- One dose (1)
- Two doses (2)

Q4 If no, will you choose to get vaccinated once it becomes available to you?

- Yes
- No

Q5 If no, what are the factors that made you decide not to get vaccinated?

- Health issues
- Previously got infected by COVID-19
- Vaccine safety concerns
- Vaccine effectiveness concerns
- Other, please specify below _____________________________________

Q6 Gender

- Male
- Female

Q7 Age Category

- 18 - 24
- 25 - 34
- 35 - 44
- 45 - 54
- 55 - 64
- 65 or older

Q8 Ethnicity

- White
- African American
- Asian
- Native American
- Other

Q9 Education: What is the highest degree or level of education completed?

- No schooling completed
- Some high school, no diploma
- High school graduate, diploma or equivalent (ex: GED)
- Some college credit, no degree
- Trade/technical/vocational training
- Associate degree
- Bachelor's degree
- Master's degree
- Doctorate degree

**Appendix 3:** Follow-up survey questions (Survey 3)

Q2 Have you changed your mind and decided to get the COVID-19 vaccine after you chose not to get vaccinated last year?

- No
- yes
- Does not apply

Q3 If yes, Have you been vaccinated yet?

- Yes
- No

Q4 What are the factors that made you change your mind and convince you to get the COVID-19 vaccine?

- Trusted the vaccine safety
- Trusted the vaccine efficacy
- The vaccine is free
- Recommended by trusted people
- It is mandatory to get vaccinated at my work
- Personal experience from someone I know who got the vaccine with no complications
- Others, please specify below ____________________________________

Q5 Are you considering accepting the vaccine at some point?

- Yes
- No

Q6 If yes, what are the factors that may convince you to accept getting vaccinated?

- Trust the vaccine safety
- Trust the vaccine efficacy
- Recommendations from trusted people
- Being mandatory at my work
- Personal experience from someone I know who got the vaccine with no complications
- Other, please specify below _______________________________________

Q7 If no, What are the factors that affect your decision not to get vaccinated?

- Health issues
- Previously got infected by COVID-19
- Vaccine safety concerns
- Vaccine effectiveness concerns
- Other, please specify below ______________________________________

Q8 Which of the following would you trust most that helped you decide whether you would get a COVID-19 vaccine?

- Health Workers
- Government or Health Department
- Family or Friends
- Famous persons or religious leaders
- News, newspapers, radio, or online groups
- Others, please specify ________________________________________

Q9 Gender

- Male
- Female

Q10 Age Category

- 18 - 24
- 25 - 34
- 35 - 44
- 45 - 54
- 55 - 64
- 65 or older

Q11 Ethnicity

- White
- African American
- Asian
- Native American
- Other

Q12 Education: What is the highest degree or level of education completed?

- No schooling completed
- Some high school, no diploma
- High school graduate, diploma or equivalent (ex: GED)
- Some college credit, no degree
- Trade/technical/vocational training
- Associate degree
- Bachelor's degree
- Master's degree
- Doctorate degree

**Appendix 4:** Side effects survey questions (Survey 4)

Q1 Have you gotten vaccinated with any doses of a COVID-19 vaccine?

- Yes
- No

Q2 If yes, which vaccine did you get?

- Pfizer-BioNTech
- Moderna
- Johnson & Johnson/ Jansen
- One dose from a company and the second from another
- Other, please specify below ____________________________________

Q3 How many doses did you get so far?

- One dose (1)
- Two doses (2)
- Three doses (3), Booster #1
- Four doses (4), Booster #2

Q4 Have you gotten infected by COVID-19 after getting vaccinated with any of the vaccine doses? (Please choose all that apply)

- No
- Yes, after the first dose (1)
- Yes, after the second dose (2)
- Yes, after the third dose (3), Booster #1
- Yes, after the fourth dose (4), Booster #2

Q5 Do you think that COVID-19 vaccines are safe in the long term?

- Definitely yes
- Probably yes
- Might or might not
- Probably not
- Definitely not

Q6 Have you noticed any symptoms following vaccination? (please choose all that apply)

- No symptoms at all
- Yes, minor/ moderate symptoms
- Yes, major symptoms

Q7 After which dose of the vaccine have you experienced these symptoms? (please choose all that apply)

- 1st dose
- 2nd dose
- 3rd dose, Booster #1
- 4th dose, Booster #2

Q8 If you chose the 1st dose, have you experienced any of the following minor/ moderate symptoms (please choose all that apply):

- Low-grade fever between 99.5°F (37.5°C) and 100.3°F (38.3°C)
- Tiredness/ Fatigue
- Pain at the injection site
- Inflammation or swelling at the injection site
- Headache
- Joints pain
- Muscular pain
- Skin rash
- Irritation or itchy skin
- Diarrhea
- Nausea/ Vomiting
- Others, please specify below ____________________________________

Q9 If you chose the 1st dose, have you experienced any of the following major symptoms (please choose all that apply):

- Anaphylaxis (a life-threatening allergic reaction)
- Anxiety
- High-grade fever >100.3°F (38.3°C)
- Dyspnea (shortness of breath)
- Loss of consciousness
- Blood clots
- Seizure
- Myocarditis (inflammation of the heart muscle)
- Hospitalization
- Others, please specify below _____________________________________

Q10 If you chose the 1st dose, have you sought any medical advice regarding these symptoms?

- Yes
- No

Q11 If you chose the 2nd dose, have you experienced any of the following minor/ moderate symptoms (please choose all that apply):

- Low-grade fever between 99.5°F (37.5°C) and 100.3°F (38.3°C)
- Tiredness/ Fatigue
- Pain at the injection site
- Inflammation or swelling at the injection site
- Headache
- Joints pain
- Muscular pain
- Skin rash
- Irritation or itchy skin
- Diarrhea
- Nausea/ Vomiting
- Others, please specify below ________________________________________

Q12 If you chose the 2nd dose, have you experienced any of the following major symptoms (please choose all that apply):

- Anaphylaxis (a life-threatening allergic reaction)
- Anxiety
- High-grade fever >100.3°F (38.3°C)
- Dyspnea (shortness of breath)
- Loss of consciousness
- Blood clots
- Seizure
- Myocarditis (inflammation of the heart muscle)
- Hospitalization
- Others, please specify below _____________________________________

Q13 If you chose the 2nd dose, have you sought any medical advice regarding these symptoms?

- Yes
- No

Q14 If you chose the 3rd dose (1st Booster dose), have you experienced any of the following minor/ moderate symptoms (please choose all that apply):

- Low-grade fever between 99.5°F (37.5°C) and 100.3°F (38.3°C)
- Tiredness/ Fatigue
- Pain at the injection site
- Inflammation or swelling at the injection site
- Headache
- Joints pain
- Muscular pain
- Skin rash
- Irritation or itchy skin
- Diarrhea
- Nausea/ Vomiting
- Others, please specify below ________________________________________

Q15 If you chose the 3rd dose (1st Booster dose), have you experienced any of the following major symptoms (please choose all that apply):

- Anaphylaxis (a life-threatening allergic reaction)
- Anxiety
- High-grade fever >100.3°F (38.3°C)
- Dyspnea (shortness of breath)
- Loss of consciousness
- Blood clots
- Seizure
- Myocarditis (inflammation of the heart muscle)
- Hospitalization
- Others, please specify below _______________________________________

Q16 If you chose the 3rd dose (1st Booster dose), have you sought any medical advice regarding these symptoms?

- Yes
- No

Q17 If you chose the 4th dose (2nd Booster dose), have you experienced any of the following minor/ moderate symptoms (please choose all that apply):

- Low-grade fever between 99.5°F (37.5°C) and 100.3°F (38.3°C)
- Tiredness/ Fatigue
- Pain at the injection site
- Inflammation or swelling at the injection site
- Headache
- Joints pain
- Muscular pain
- Skin rash
- Irritation or itchy skin
- Diarrhea
- Nausea/ Vomiting
- Others, please specify below _______________________________________

Q18 If you chose the 4th dose (2nd Booster dose), have you experienced any of the following major symptoms (please choose all that apply):

- Anaphylaxis (a life-threatening allergic reaction)
- Anxiety
- High-grade fever >100.3°F (38.3°C)
- Dyspnea (shortness of breath)
- Loss of consciousness
- Blood clots
- Seizure
- Myocarditis (inflammation of the heart muscle)
- Hospitalization
- Others, please specify below ___________________________________

Q19 If you chose the 4th dose (2nd Booster dose), have you sought any medical advice regarding these symptoms?

- Yes
- No

Q20 Gender

- Male
- Female

Q21 Age Category

- 18 - 24
- 25 - 34
- 35 - 44
- 45 - 54
- 55 - 64
- 65 or older

Q22 Ethnicity

- White
- African American
- Asian
- Native American
- Other

Q23 Education: What is the highest degree or level of education completed?

- No schooling completed
- Some high school, no diploma
- High school graduate, diploma or equivalent (ex: GED)
- Some college credit, no degree
- Trade/technical/vocational training
- Associate degree
- Bachelor's degree
- Master's degree
- Doctorate degree

**Table S1:**

Response rates between different groups of participants among the 4 surveys. The participants’ groups are University faculty, students, and employees (MS), University-affiliated Health System registered patients (MP), and University-affiliated Comprehensive Cancer Center registered cancer patients (MCP).

|  | **Pre-vaccination Survey**  (Survey 1) | **Post-vaccination Survey**  (Survey 2) | **Follow-up Survey**  (Survey 3) | **Adverse Events Survey**  (Survey 4) |
| --- | --- | --- | --- | --- |
|  | n *%* | n *%* | n *%* | n *%* |
| **MS (N=18,206)** | 4,983 *27.4* | 3,587 *19.7* | 2,995 *16.5* | 1,917 *10.5* |
| **MP (N=126,728)** | 9,551 *7.5* | 7,189 *5.7* | 3,485 *2.7* | 4,400 *3.5* |
| **MCP (N=12,358)** | 827 *7* | 763 *6.2* | 422 *3.4* | 459 *3.7* |
| **Total (N=157,292)** | 15,361 *10* | 11,539 *7.3* | 6,902 *4.3* | 6,776 *4.3* |

*N= Number of targeted participants

**Table S2.**

Risk factors for COVID-19 vaccination hesitance in four consecutive surveys: Survey 1(Pre-vaccination), Survey 2 (Post-vaccination), Survey 3 (Follow-up), and Survey 4 (Side effects).

|  | Survey 1 | | Survey 2 | | Survey 3 | | Survey 4 | |
| --- | --- | --- | --- | --- | --- | --- | --- | --- |
|  | **Univariate** | **Multivariate** | **Univariate** | **Multivariate** | **Univariate** | **Multivariate** | **Univariate** | **Multivariate** |
| **Age** | p<0.05^*^ | p<0.05^*^ | p<0.001^+^ | p<0.001^+^ | N.S. | P<0.001 | p<0.001 | p<0.001 |
| **Gender** | N.S. | N.S. | p<0.001 | N.S. | p<0.05 | N.S. | p<0.001 | p<0.001 |
| **Level of Education** | p<0.05 | P<0.05 | p<0.001 | p<0.001 | N.S. | N.S. | p<0.001 | p<0.001 |
| **Race/Ethnicity (White)** | p<0.05 | N.S. | N.S. | N.S. | N.S. | N.S. | N.S. | N.S. |
| **Concerns with the Vaccine** | N.S. | N.S. | N.S. | N.S. | N.S. | N.S. | N/A | N/A |
| **No Health Problems (*vs.* patient)** | p<0.05 | p<0.05 | N/A | N/A | N/A | N/A | N/A | N/A |
| **Trusted Sources (Survey 3)** | N/A | N/A | N/A | N/A | p<0.001 | p<0.05 | N/A | N/A |
| **Type of Vaccine (Survey 4)** | N/A | N/A | N/A | N/A | N/A | N/A | p<0.001 | p<0.001 |

N.S.: Not Significant. N/A: Non-applicable. ^*^(>44 years). ^+^(>34 years).

**Figure S1:**

Trusted Sources convincing participants to get vaccinated (survey 3). The groups of the participants are University faculty, students, and employees (MS), University-affiliated Health System registered patients (MP), and University-affiliated Comprehensive Cancer Center registered cancer patients (MCP). Nearly three-quarters of the participants trusted healthcare workers (46) and their families and friends (27) with their recommendations in accepting the COVID-19 vaccination.
